# Homogeneous Spatial Distribution of Deuterium Chemisorbed on Free-Standing Graphene

**DOI:** 10.3390/nano12152613

**Published:** 2022-07-29

**Authors:** Maria Grazia Betti, Elena Blundo, Marta De Luca, Marco Felici, Riccardo Frisenda, Yoshikazu Ito, Samuel Jeong, Dario Marchiani, Carlo Mariani, Antonio Polimeni, Marco Sbroscia, Francesco Trequattrini, Rinaldo Trotta

**Affiliations:** 1 INFN Sezione di Roma 1, Sapienza Università di Roma, P.le Aldo Moro 2, 00185 Rome, Italy; 2 Dipartimento di Fisica, Sapienza Università di Roma, P.le Aldo Moro 2, 00185 Rome, Italy; elena.blundo@uniroma1.it (E.B.); marta.deluca@uniroma1.it (M.D.L.); marco.felici@roma1.infn.it (M.F.); dario.marchiani@uniroma1.it (D.M.); antonio.polimeni@roma1.infn.it (A.P.); marco.sbroscia@uniroma1.it (M.S.); francesco.trequattrini@roma1.infn.it (F.T.); rinaldo.trotta@uniroma1.it (R.T.); 3 Institute of Applied Physics, Graduate School of Pure and Applied Sciences, University of Tsukuba, Tsukuba 305-8573, Japan; ito.yoshikazu.ga@u.tsukuba.ac.jp (Y.I.); jeong.samuel.fp@u.tsukuba.ac.jp (S.J.)

**Keywords:** nanoporous graphene, deuterium, graphane, XPS, Raman

## Abstract

Atomic deuterium (D) adsorption on free-standing nanoporous graphene obtained by ultra-high vacuum D${}_{2}$ molecular cracking reveals a homogeneous distribution all over the nanoporous graphene sample, as deduced by ultra-high vacuum Raman spectroscopy combined with core-level photoemission spectroscopy. Raman microscopy unveils the presence of bonding distortion, from the signal associated to the planar sp${}^{2}$ configuration of graphene toward the sp${}^{3}$ tetrahedral structure of graphane. The establishment of D–C sp${}^{3}$ hybrid bonds is also clearly determined by high-resolution X-ray photoelectron spectroscopy and spatially correlated to the Auger spectroscopy signal. This work shows that the low-energy molecular cracking of D${}_{2}$ in an ultra-high vacuum is an efficient strategy for obtaining high-quality semiconducting graphane with homogeneous uptake of deuterium atoms, as confirmed by this combined optical and electronic spectro-microscopy study wholly carried out in ultra-high vacuum conditions.

## 1. Introduction

Graphene (Gr) is at the forefront of cutting-edge science and technology. The conversion of semimetallic graphene into a semiconducting phase would open new perspectives for electronic devices and would provide new opportunities for large band gap semiconducting graphene-based photonics. The considerable effort on the chemical functionalization of graphene to induce a gap opening can entail the covalent modification of its planar carbon mesh. Atomic hydrogen/deuterium interaction at the basal plane of graphene breaks its local π structure with the sp${}^{2}$ hybridization and distorts the surrounding lattice toward a tetrahedral configuration with each carbon atom in the honeycomb lattice bound to hydrogen/deuterium, alternately up and down in the ideal graphane configuration [1,2,3,4,5,6,7].

Although graphene has high thermodynamic stability and chemical inertness, it can be patterned [8,9] or decorated with nanoparticles [10,11] or molecules [12], to build up sensors. However, a different covalent bonding in a distorted lattice can be hindered by a high kinetic barrier associated with the rearrangement of the planar carbon lattice. Furthermore, a bonding modification of graphene into a tetrahedral geometry due to a hydrogenation/deuteration process can induce defects, grain boundaries, vacancies, and implanted atoms. The amount and nature of defects depend both on the hydrogenation/deuteration methods and on the homogeneity of the pristine carbon planar structure.

Several methods have been employed to hydrogenate Gr, ranging from hot [3,13,14] to cold plasma [15,16] deposition, to very low-energy ion exposure [5,6], to molecular H${}_{2}$ high-temperature cracking [7,17,18], aimed at achieving a high hydrogen uptake, while minimizing undesired side reactions or irreversible defects/damage of the carbon lattice. When converting two-dimensional (2D) layer(s) of semimetallic Gr into an ideal semiconducting graphane configuration upon atomic hydrogen/deuterium chemisorption, it is therefore of fundamental importance to be able to carefully probe the evolution of defects in order to establish their precise nature and influence on the graphane properties. It is worth noting that defects, even with low density, can play a crucial role in the chemical reactivity, as they can introduce electronic states in the energy gap and/or influence the electron mobility.

We herewith expose to atomic deuterium a three-dimensional (3D) warped free-standing 2D graphene, constituted by a compact, bi-continuous interconnected 3D arrangement of high-quality graphene veils, composed by one to two layers, weakly interacting among them [19,20]. The topology of this nanoporous graphene (NPG) with intrinsic curvatures and rippling at the μm scale can foster H and D adsorption. In fact, it has been shown that the energy barrier for H/D chemisorption on graphene decreases [21] thanks to the pullout of the C atom toward the proton or deuteron to form the sp${}^{3}$ bond [22], as observed in recent experiments [5,6]. NPGs are defect-free graphene specimens with very high specific surface areas [23,24], where chemisorbed hydrogen or deuterium atoms form a thermodynamically stable configuration, mimicking the ideal graphane structure [5,7]. In this work, highly controlled deuteration of NPG specimens is obtained by D${}_{2}$ molecular cracking in ultra-high vacuum (UHV) conditions to achieve a very low-damage deposition for long time exposures, ensuring a good D uptake, and a low presence of any other lattice defects than the establishment of pure sp${}^{3}$ bonds [7].

We investigate pristine and deuterated NPG by Raman and photoelectron spectroscopies. Raman spectroscopy is a well-known sensitive technique to probe disorder and defects in graphene through defect-associated peaks. In fact, the Raman-forbidden D and D${}^{\prime }$ bands are activated by a single-phonon intervalley and intravalley scattering process, respectively. Defects, even at low density, provide the missing momentum to satisfy momentum conservation in those Raman scattering processes. Furthermore, the D and D${}^{\prime }$ Raman peak intensities are related to the density of defects, and their intensity ratio can give insights on the nature of the defects, as clearly demonstrated in several theoretical and experimental Raman results [25,26,27]. A direct proof of sp${}^{3}$ C-D (C-H) bonding formation, and also of the possible presence of dangling bond defects, can be obtained by X-ray photoelectron spectroscopy (XPS), through measurements of the C 1s core-level components [28,29,30].

Here, we present a spatially resolved Raman spectro-microscopy experiment in an innovative ultra-high vacuum setup, also hosting high-resolution X-ray photoelectron spectroscopy. We show that this combined optical and electronic spectroscopy approach allows us to demonstrate the almost defect-free configuration of pristine free-standing graphene as well as the highly homogeneous uptake of deuterium atoms, which leads to a significant lattice distortion from the planar C mesh toward the sp${}^{3}$ hybridized configuration, with a negligible formation of vacancies, dangling bonds, and other lattice defects. A conclusive confirmation of these findings is achieved by XPS spectro-microscopy, where the C 1s core level discriminates between components, either due to vacancies/dangling bonds or induced by sp${}^{3}$ bonding. Finally, a correlation between spatially resolved core-level photoemission and Auger spectroscopy further upholds the local formation of the sp${}^{3}$ hybridized configurations at the micrometer scale in these deuterated-NPG samples.

The disentanglement of the influence of sp${}^{3}$ carbon bonds from lattice, edge, and unsaturated bond defects can be achieved thanks to the combined use of optical (Raman) and electronic (XPS) spectro-microscopies, and exposure of fully free-standing graphene to highly efficient molecular cracked D${}_{2}$ in UHV. These adsorption procedures define a viable and clean method for the conversion of graphene into semiconducting graphane, also opening the way to further sp${}^{3}$-Gr functionalization towards novel advanced devices [31,32].

## 2. Materials and Methods

The nanoporous graphene samples were synthesised by employing a nanoporous Ni-based chemical vapour deposition (CVD) method [33,34,35,36,37]. Ni${}_{30}$Mn${}_{70}$ ingots were grown by melting the two pure metals (>99.9 at.% purity) in an Ar-protected arc melting furnace. These as-prepared Ni${}_{30}$Mn${}_{70}$ ingots were then annealed at 900 ${}^{\circ }$C for one day to obtain a composition homogeneous microstructure. They were afterwards cold-rolled to 50 μm-thin sheets at room temperature. The nanoporous Ni was obtained from the so achieved Ni${}_{30}$Mn${}_{70}$ sheet by using chemical dealloying in a 1.0 M (NH${}_{4}$)${}_{2}$SO${}_{4}$ aqueous solution at 50 ${}^{\circ }$C lasting 12 h. These dried and cleaned nanoporous Ni substrates were loaded on a corundum plate, and treated for reduction treatment; they were located into the center of a quartz tube (ϕ30 ×ϕ30 × 1000 mm) and annealed at 900 ${}^{\circ }$C under flowing gas, constituted by 200 sccm Ar (purity 99.999%) and 100 sccm H${}_{2}$ (purity 99.999%) for 3 min. The CVD process to obtain graphene used benzene (0.5 mbar, 99.8%, anhydrous, Sigma Aldrich, Darmstadt, Germany) as a precursor, together with gas flow of Ar (200 sccm) and H${}_{2}$ (100 sccm), at 800 ${}^{\circ }$C for 120 s. After graphene growth, the furnace was opened and the quartz tube was quickly cooled to room temperature. These nanoporous Ni substrates were then dissolved in a solution (1.0 M) of HCl at 25 ${}^{\circ }$C for 12 h, and afterwards transferred into another HCl solution (2.0 M at 60 ${}^{\circ }$C) to further remove residual Ni and Mn. Finally, the NPG samples were repeatedly washed in distilled water and kept in solution for one day, then transferred into isopropanol (99.7%, Kanto Chemical Co., Inc., Tokio, Japan) and kept for one week. As final step, the nano–porous graphene sheets were dried by using CO${}_{2}$ gas (98% purity).

Pristine NPG cleaning in UHV has been carried out by high-temperature annealing (up to ∼620 ${}^{\circ }$C), as described in detail elsewhere [6]. Pristine cleaned NPG has been exposed to a focused beam of atomic D with a total exposure of 26.1 × 10${}^{3}$ Langmuir, namely for 9 h 40 min at 1 × 10${}^{-6}$ mbar (recalling that 1 Langmuir= 1 s × 10${}^{-6}$ Torr). Atomic deuterium has been produced by cracking the D${}_{2}$ molecules in UHV and letting atomic deuterium flow directly onto the NPG sample. A molecular cracker in UHV was used (Focus GmbH, Hunstetten, Germany EFM-H atomic hydrogen source) constituted by a tungsten capillary positioned at a few cm from the NPG sample, heated at approximately 2400 ${}^{\circ }$C, where the clean flow of 5N molecular D${}_{2}$ is cracked into atoms. With this method, more than 95% molecular cracking efficiency has been demonstrated [38]. As a consequence, the beam of atomic D flushes onto NPG with an average energy of about 0.22 eV, and D atoms directly bind to the C atoms in the graphene mesh. The long exposure times and the use of neutral D atoms enable a more efficient deuteration process with respect to previous works [5,6], with deuterium flowing through the pores in depth in the NPG specimens. In fact, such long exposure time, well beyond the saturation of the sp${}^{3}$ component in the C1s XPS spectra ensures a deep penetration of the D atoms in the whole NPG samples, inducing intense Raman D bands.

The Raman, XPS, and Auger Electron Spectroscopy experiments were performed at Sapienza University in Rome, in the SMART laboratory of the Department of Physics. This is a newly conceived, integrated experimental apparatus, constituted by three UHV-interconnected chambers in a novel set-up recently designed and assembled to combine optical and electron micro-spectroscopy all in UHV. The three vacuum chambers, hosting the micro-XPS, micro-Raman and preparation stages, respectively, had base pressures in the high 10${}^{-11}$ mbar range. For the XPS measurements, X-rays were generated by an Al K${}_{\alpha }$ monochromatic (1486.6 eV) source (SPECS XR50 MF) with focused beam, and the photoelectrons were analyzed by a SPECS PHOIBOS 150 analyzer from SPECS group, Berlin, Germany with energy resolution of ∼0.4 eV. The ultimate XPS spatial resolution was 30 μm. The binding energy (BE) scale was calibrated on a freshly sputtered gold foil in electrical contact with the sample. The C KVV Auger signal, produced by the intrinsic de-excitation process after photoionization of the C 1s core level, was taken from the survey XPS spectrum spanning to low kinetic (high binding) energy, by using 20 eV pass energy. The Auger integral signal N(E) has been smoothed by the Savitzky–Golay method and numerically differentiated to obtain the typical first derivative dN(E)/dE spectrum.

The Raman experiments were carried out in the novel UHV setup in a backscattering configuration, by using as excitation source a single frequency Nd:YVO${}_{4}$ laser at 532.2 nm. The laser and the signal were sent through a customized 60× objective (NA = 0.82) compatible with UHV chambers. The laser power was kept below 200 μW to avoid sample damage. A 50-cm focal length monochromator equipped with a 300 or 1200 grooves/mm grating was employed to spectrally analyse the signal, which was detected by a back-illuminated liquid $N_{2}$-cooled Si CCD camera. Rejection of laser light was achieved on the excitation path by using a wavelength-selective beam splitter that cuts only the 532-nm light, and on the detection path by using a sharp super-notch filter for 532-nm light. The Raman sample holder, mounted onto a hexapode piezoelectric stage can be moved with steps as small as 50 nm. The overall setup spatial resolution was approximately 500 nm (limited by the laser source wavelength and focal spot).

NPG samples have been imaged by scanning electron microscopy (SEM) by using the apparatus of the CNIS laboratory at Sapienza University: field-emission Zeiss Auriga 405, nominal resolution of 1 nm at maximum magnification, beam energy of 9.5 keV and working distance of approximately 3.5 mm.

## 3. Results and Discussion

One of the most effective spectroscopy techniques for determining the thickness, doping, strain, lattice deformation, and defects in 2D materials is the Raman technique, widely used in graphene [25,39,40], in nanoporous graphene [19,20], and other 2D systems [41,42,43,44,45,46,47].

**Figure 1:**
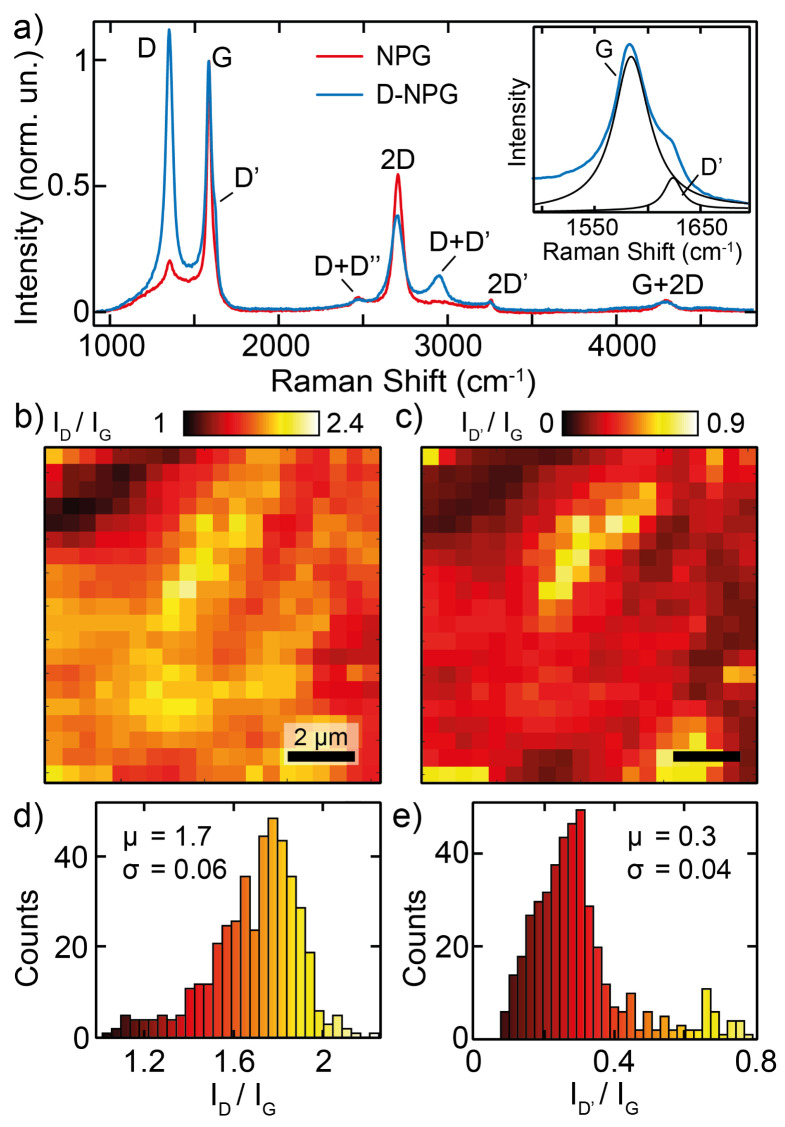
(**a**) Raman spectra taken on the pristine (red line) and deuterated NPG (blue line) sample, acquired with a λ = 532.2 nm excitation laser; in the inset we see the zoomed region of G and D′ bands with Lorentzian fitting. (**b**,**c**) Deuterated-NPG: spatial mappings of the I${}_{D}$/I${}_{G}$ and I${}_{D\prime }$/I${}_{G}$ intensity ratios, respectively, over an area of 10 μm × 10 μm with a 500-nm step. (**d**,**e**) Deuterated-NPG: occurrence distribution of the I${}_{D}$/I${}_{G}$ and I${}_{D\prime }$/I${}_{G}$ intensity ratios, respectively (μ, mean value; σ, standard deviation).

The Raman spectra taken on the pristine and deuterated-NPG samples in UHV ambient are shown in Figure 1a. The Raman spectrum of pristine NPG, after an annealing at 610 ${}^{\circ }$C in UHV for about 1 h, shows two dominant peaks, assigned to the G and 2D bands located at 1585 and 2701 cm${}^{-1}$, respectively, typical of high-quality graphene samples. The low-intensity D band is slightly visible due to the presence of a tiny defect density, and the two-phonon bands (2D′, D + D′, and D + D″) are also detectable. The low intensity of these peaks in the pristine sample indicates a low density of lattice defects and/or vacancies and/or contaminants, and the absence of amorphous phases. On the other hand, the Raman spectrum of the deuterated NPG (normalized to the pristine NPG spectrum by using the G band intensity) displays a prominent D peak and a lower intensity associated to the 2D band. The atomic deuterium chemisorption induces the reduction of the G and 2D peaks, due to the symmetry breaking of the planar sp${}^{2}$ configuration, whereas the D, D′, and D + D′ increase the intensity (see the inset of Figure 1a for the D′ peak). Why do these plethora of Raman lines differently respond to the deuteration process? It is worth recalling that defects/disorder cause a linear increase of the γ width for both the two-phonon process lines (2D, 2D′ and D + D″) and those activated by defects (D and D′, and D + D′). Whereas the 2D, 2D′, and D + D″ lines depend on the density of defects $n_{d}$ only through the electronic linewidth γ, on the other hand for the defect-induced D, D′, and D + D′ lines the intensity also increases as a function of $n_{d}$, as discussed in detail in the theoretical description of double-resonant Raman spectra [26]. In particular, the 2D band increases its full width half maximum by 30%, and the D′ intensity increases its intensity by more than one order of magnitude.

The increased intensity of the D and D′ bands can be due to different type of effects in the graphene lattice, such as sp${}^{3}$ bonds, C vacancies, or contaminants [27]. In the present case where deuteration of free-standing nanoporous graphene has been performed by long exposure times to low-energy atomic D, the D atoms diffuse into the pores and penetrate deep into the whole NPG samples, inducing intense Raman D bands, whose large intensity can be mainly ascribed to the distortion of sp${}^{2}$ bonds into sp${}^{3}$ tetrahedral configuration at the C-D bonding sites [15]. Furthermore, the intrinsic curvature of the porous structure of NPG favors the establishment of the non-planar sp${}^{3}$ bond by lowering the chemisorption energy barrier, as shown for atomic hydrogen on graphene [21,22,48,49,50].

**Figure 2:**
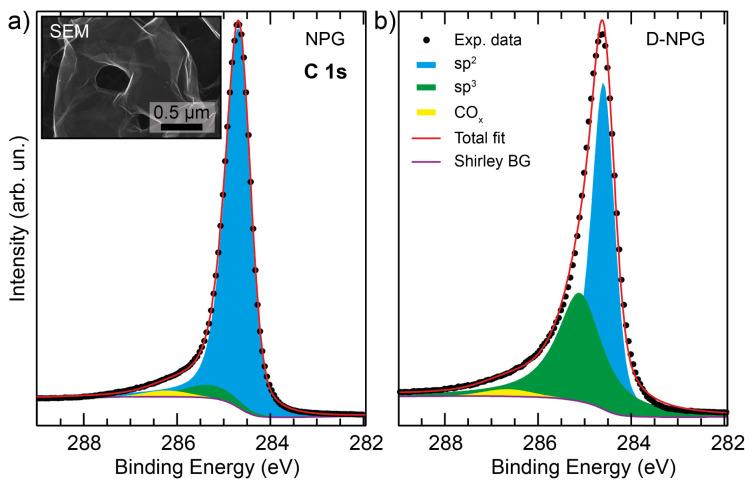
C 1s core level XPS spectra and fitting curves for clean pristine NPG (**a**) and deuterated-NPG (**b**); experimental data (black dots), sp${}^{2}$ fitting component (blue areas), sp${}^{3}$ component (green areas), CO${}_{x}$ component (yellow areas), Shirley background (violet lines), and fitting sum curve (red lines). An SEM image of NPG is reported in the inset to panel (**a**).

In order to probe the homogeneity of the deuteration process, Raman spectra of deuterated NPG have been acquired over a region of 10 × 10 μm${}^{2}$, sampled in steps of 500 nm. We remark that such a spatial step compares with the laser excitation wavelength and focused spot area on the sample, thus constituting the best achievable spatial resolution. From the Raman spectral data in each pixel, we perform a Lorentzian fit of the D, G, and D′ peaks, whose results are used to plot the spatial mappings of the I${}_{D}$/I${}_{G}$ and I${}_{D\prime }$/I${}_{G}$ Raman band-relative intensity ratios over the sampled area, as shown in Figure 1b,c, respectively. The intensity ratio occurrences as derived from the mappings are displayed as histograms in panels d,e. The D and D′ band normalised intensity maps present an analogous spatial distribution across the sampled area, which closely resembles the topography of the SEM image (see inset in Figure 2) of the nanoporous structure of NPG. The I${}_{D}$/I${}_{G}$ distribution presents a rather homogeneous distribution, where the normalised intensity range is centered at 1.7 with a narrow distribution, whereas the I${}_{D\prime }$/I${}_{G}$ distribution centered at 0.3 presents a slightly wider relative shape. The former distribution is associated with the presence of atomic deuterium chemisorbed on the C lattice. On the other hand, the distribution of the I${}_{D\prime }$/I${}_{G}$ can be associated with the presence of different type of defects, among them the sp${}^{3}$ non-planar bonds associated to the D-C bondings. It is worth noting that in several areas of the sampled mapping, the I${}_{D}$/I${}_{D\prime }$ ratio is of the order of 10-to-13, which is well known to be associated to the transition from the sp${}^{2}$ to the sp${}^{3}$ bonds [26,27], although we cannot neglect the presence of a small density of lattice defects with different nature, not only due to the sp${}^{3}$ distorted lattice. The investigation of the spatial distribution of the different type of defects is beyond the scope of this paper, and it deserves further detailed theoretical and experimental investigations.

A further proof that the exposure to atomic D leads to the establishment of lattice distortion associated with non-planar D-to-C bonds, and also to the formation of other lattice defects, can be obtained by the analysis of the C 1s core level by XPS. The C 1s core level spectra for pristine clean NPG and deuterated NPG are shown in Figure 2. Experimental data have been fitted with pseudo-Voigt (Gaussian and Lorentzian) curves, considering the overall experimental resolution (Gaussian) and the intrinsic lineshape (Lorentzian), after subtraction of a Shirley background, according to well-established procedures [6,28].

Pristine clean NPG is dominated by the huge, narrow, and slightly asymmetric sp${}^{2}$ component [28] at 284.7 eV BE. There is a small sp${}^{3}$-like component due to the bent and wrinkled regions of NPG [19,20] and a tiny peak due to residual (less than 2%) CO${}_{x}$ components [51,52,53].

In the C 1s core level of deuterated NPG, the sp${}^{3}$ component noticeably increases, marking the establishment of D-to-C non-planar bonds [5,6]. The estimation of the average deuterium uptake Θ can be obtained by measuring the relative intensity of the sp${}^{3}$-related component (Θ = I(sp${}^{3}$)/[I(sp${}^{2}$) + I(sp${}^{3}$)]), which is directly associated with the deuterium chemisorption on the C atoms. We obtain a concentration of about 40 at.% of atomic deuterium on carbon, comparable to the concentration achieved in previous experiments where low-energy deuterium ions were used [5,6]. It is also worth noticing the absence of any other C 1s components. In particular we do not observe in the lower BE range any peak due to unsaturated dangling bonds typical of vacancy sites [29,30,54,55,56,57]. We also recall the Raman band spectral ratios that indicate a homogeneous deuterium bonding to carbon, in agreement with the XPS data. This evidence demonstrates that the present method employing low-energy (∼0.2 eV) atomic deuterium exposure in UHV, albeit not obtaining larger concentrations of chemisorbed deuterium with respect to previous works [5,6], is nondestructive, does not produce general lattice defects, and homogeneously causes the expected sp${}^{3}$ bond formation at each C-D site.

**Figure 3:**
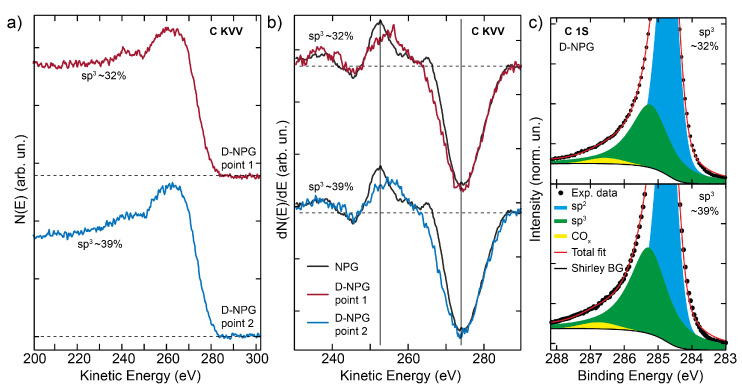
(**a**) Carbon KVV N(E) Auger electron spectroscopy peak taken on two different spatial points of the deuterated NPG (blue and red lines). (**b**) Numerically derived first derivative [dN(E)/dE] of the C KVV Auger signal from panel (**a**) compared with the derivative of the Auger signal for the pristine NPG (black lines). (**c**) C 1s core-level XPS spectra in a zoomed binding energy region and fitting curves, taken in the same spatial points of the deuterated NPG; experimental data (black dots), sp${}^{2}$ fitting component (blue areas), sp${}^{3}$ component (green areas), CO${}_{x}$ component (yellow areas), Shirley background (black lines), and fitting sum curve (red lines).

So far we have found that Raman and core-level photoemission confirm the establishment of sp${}^{3}$ hybridized bonds, the tiny presence of other lattice defects, and the rather homogeneous atomic D-C bonding at the micrometer scale of the present free-standing graphane. To further confirm the unequivocal fingerprint of sp${}^{3}$ bond formation, we can also look at the Auger spectroscopy signal. Auger has the potential to provide bonding information on carbon materials [58,59], in particular the C KVV Auger line shape should reflect the type of C-metal bonding (carbidic dominated by sp${}^{3}$ configuration or graphitic with sp${}^{2}$ lattice configuration). The Auger peaks of two representative spatially resolved (50 μm) C KVV Auger lines for the deuterated NPG, are reported as integral signal N(E) and as numerically performed first derivative data dN(E)/dE (Figure 3a and Figure 3b, respectively). The first derivative Auger lineshape of pristine graphene (black lines in Figure 3b) is characterized by the pronounced minimum at 272 eV kinetic energy (KE) and by the small sharp positive peak at about 265 eV KE; all these signatures are associated to the dominant sp${}^{2}$ bonds [58]. Upon deuterium uptake, the lineshape is modified, with a small reduction of the small positive peak, and a lower energy difference between the highest positive and lowest negative wings of the dN(E)/dE signal. These represent the hallmarks of a transition from a pure sp${}^{2}$ graphitic Auger signal to a higher sp${}^{3}$ content [58,59]. It is worth noting that the C KVV Auger lineshape shows the co-existence of sp${}^{3}$ and sp${}^{2}$ bonds, still present due to the actual atomic deuterium percentage adsorbed on top of the C atoms.

The Auger and the C1s core levels taken in different spatial points with resolution of 50 μm unveil a homogeneous spatial distribution of atomic deuterium over the whole NPG sample. However, whereas the C 1s core-level spectra for deuterated NPG measured in different points reveal slight differences in deuterium content (∼32 at.% and ∼39 at.%, as shown in Figure 3c), the spatially corresponding Auger lineshapes (Figure 3a,b) taken in the same spatial points of the XPS are less sensitive to these subtle differences in deuterium uptake. The slightly higher deuterium content accompanied by a more intense sp${}^{3}$ component in the XPS lineshape is only accompanied by a slight change of the Auger signal slope.

## 4. Conclusions

The chemisorption of atomic deuterium on the C lattice of free-standing nanoporous graphene, has been determined by using complementary all-in-UHV optical (Raman) and electronic (XPS) spatially resolved spectroscopies, combined in a new and original micro-spectroscopy setup. The use of a high-temperature D${}_{2}$ molecular cracker directed onto the NPG sample in an ultra-high vacuum allows us to obtain high-quality and homogeneous distribution of deuterium atoms covalently bound to carbon. In particular, spatially resolved Raman maps unveil a rather homogeneous atomic deuterium uptake. The lattice distortion due to the sp${}^{3}$ bond deformation from the pristine sp${}^{2}$ planar bonds is brought to light by Raman spectro-microscopy, though the formation of a tiny density of defects cannot be excluded. Clear and further fingerprints of the achievement of the sp${}^{3}$ bonds between the deuterium and the carbon mesh are its detection as component in the C 1s XPS core level and the Auger KVV lineshape. This evidence, obtained with complementary optical and electronic spectroscopies all-in-UHV, demonstrates the transition from graphene with pure sp${}^{2}$ planar bonds to the formation of a homogeneous distribution of D atoms on top of the C atoms in the mesh with sp${}^{3}$ bonds, paving the way to an optimal strategy for achieving almost ideal graphane on a large spatial scale with a low density of defects.

## Data Availability

The data presented in this study are available on request from the corresponding authors.

## Abbreviations

The following abbreviations are used in this manuscript:

XPS	X ray Photoelectron Spectroscopy
NPG	Nano Porous Graphene
UHV	Ultra High Vacuum
3D	Three Dimensional
2D	Two Dimensional
